# *Brucella canis* in Commercial Dog Breeding Kennels, Ontario, Canada

**DOI:** 10.3201/eid2612.201144

**Published:** 2020-12

**Authors:** J. Scott Weese, Kathleen Hrinivich, Maureen E.C. Anderson

**Affiliations:** University of Guelph, Guelph, Ontario, Canada (J.S. Weese);; Animal Hospital of Cambridge, Cambridge, Ontario (K. Hrinivich);; Ministry of Agriculture, Food and Rural Affairs, Guelph (M.E.C. Anderson)

**Keywords:** zoonoses, brucellosis, Brucella canis, dogs, bacteria, kennels, Ontario, Canada

## Abstract

We evaluated the prevalence of *Brucella canis* seropositivity in a convenience sample of dogs from commercial breeding kennels in Ontario, Canada. Overall, 127/1,080 (11.8%) dogs from 23/63 (37%) kennels were seropositive. The prevalence of positive dogs within kennels with >1 positive dog ranged from 3.9% to 100% (median 33%).

*Brucella canis* is a dog-adapted *Brucella* species that most commonly causes reproductive disease and diskospondylitis in dogs and can be carried long-term and subclinically. Zoonotic infections are uncommonly reported (*1**–**4*), but may be underdiagnosed (*3**,**5*).

In Canada, *Brucella canis* has been found predominantly in imported dogs. However, it was identified in 2 adult female dogs from a commercial breeding kennel in Ontario, Canada, in March 2019. We conducted an investigation of prevalence and distribution of *B. canis* in the broader commercial dog breeding population.

We collected serum samples from a convenience sample of dogs at commercial breeding kennels in southern Ontario, Canada. We used rapid slide agglutination test (RSAT) and followed up with positive results using 2-mercaptoethanol RSAT (2ME-RSAT). We performed PCR on whole (EDTA treated) blood from a subset of *B. canis*−seropositive dogs. 

We identified positive RSAT and 2ME-RSAT tests in 127/1,080 (11.8%) clinically normal dogs from 23/63 (37%) kennels during March 15−December 18, 2019 (1–61 dogs/kennel, median 7). We considered reactive an additional 82 (7.6%) dogs that were positive by RSAT but negative by 2ME-RSAT; 63 (77%) of those were from kennels from which positive dogs were identified. The prevalence of positive dogs within kennels that had >1 positive dog ranged from 3.9% to 100% (median 33%). Whole blood samples from 20 dogs tested by PCR were all negative. We retested 130 dogs 4–6 weeks after the initial test (Table).

**Table:** Initial and follow-up serologic testing for *Brucella canis* in 130 dogs, Canada

Initial result	No.	Follow-up result*	No. (%)
Negative	84	Positive	0 (0)
		Reactive	0 (0)
		Negative	84 (100)
Positive	9	Positive	6 (67)
		Reactive	1 (11)
		Negative	2 (22)
Reactive	37	Positive	1 (0.3)
		Reactive	2 (0.5)
		Negative	34 (92)

*Follow-up testing was performed 4–6 weeks after the initial test.

The seropositive rate contrasts with a 1980 study that reported 0.3% seroprevalence in dogs from southwestern Ontario (*6*). Studies in other regions have reported seroprevalence rates of 0%–4.6%; higher rates (e.g., 20%–83%) were reported in some breeding kennels (*1**,**7**–**9*). A structured approach to enrollment was not possible because enrollment was based on kennel operators’ willingness to participate. Various population enrollment biases might be present in the prevalence estimate. These results should be taken as an indication of widespread presence of *B. canis* bacteria in this population, with high rates in some kennels and the potential for introduction of infected puppies into households.

Because *B. canis* infection is a notifiable disease in Ontario, we obtained data from 2013–2018 from the Ontario Ministry of Agriculture Food and Rural Affairs. Provincially, there were no positive test results for *B. canis* in dogs in 2013, 2015 and 2017, and 1–3 cases in each of 2014, 2016, and 2018. Because prior surveillance was limited, it is unclear whether this is a new problem or one that was previously overlooked. However, these 0–3 diagnoses/year and anecdotal data about recent reproductive disease in some affected kennels make it unlikely that *B. canis* infection was present but undiagnosed. The origin of the infection could not be properly investigated, but it was suspected to have originated from breeding dogs imported from eastern Europe in 2018.

Without a standard approach for clinical or surveillance testing for *B. canis* bacteria, we used the sensitive RSAT followed by the more specific 2ME-RSAT, which is considered a confirmatory test (*10*). Cases with RSAT-positive but 2ME-RSAT−negative results were common; most were subsequently negative. A possible cause was transient cross-reaction with *Bordetella bronchiseptica* vaccination or another pathogen; we could not investigate specifically because information about *B. bronchiseptica* vaccination or infection in these dogs was not available. The potential for false-positive results should be considered, particularly because infected dogs are often euthanized in accordance with regulatory requirements.

Limited PCR testing was performed because of negative results in the first 20 samples; negative results were presumed due to the intermittent nature of *B. canis* bacteremia in clinically normal animals. Although PCR or culture can provide a definitive diagnosis, sensitivity can be low for screening; it is higher when testing reproductive or fetal fluids or tissues from abortions or stillbirths for *B. canis.*

Limited clinical data were available. Some affected kennels reported substantial reproductive challenges presumably associated with brucellosis (e.g., small litter sizes, abortions, stillbirths, low conception rates) whereas no problems were reported in others. Whether this reflects lack of recognition of problems, subclinical infection, or early infection that had not yet resulted in overt reproductive disease is unclear.

Underdiagnosis of *B. canis* as a cause of nonspecific disease (e.g., undulating fever, fatigue, headache, malaise, chills, weight loss, hepatomegaly, splenomegaly, lymphadenopathy) and human brucellosis is a concern; physicians are more likely to perform serologic tests that target smooth *Brucella* species (*B. abortus, B. suis*, and *B. melitensis*). Physicians should consider the potential presence of *B. canis* in patients with disease suggestive of brucellosis, especially those with animal contact, and realize the limitations of serologic testing.

*Brucella canis* should be considered endemic to commercial dog kennels in Ontario, with potential human health risks. *B. canis* screening of breeding dogs is recommended (*10*), and testing of puppies from parents of unknown *Brucella* status is reasonable.
